# Differences in Player Performance and Longevity After Achilles Tendon Rupture Between Professional Basketball Players in the NBA and WNBA

**DOI:** 10.1177/23259671231212479

**Published:** 2024-10-04

**Authors:** David A. Momtaz, Abdullah Ghali, Farhan Ahmad, Rishi Gonuguntla, Rebecca J. Wang, Reid Yanney, Ashley Lao, Varun Bora, Theodore B. Shybut

**Affiliations:** * Department of Orthopaedics, Baylor College of Medicine, Houston, Texas, USA; Investigation performed at Department of Orthopedic Surgery, Baylor College of Medicine, Houston, Texas, USA

**Keywords:** Achilles tendon rupture, basketball, return to play, sex-based differences, NBA, WNBA

## Abstract

**Background:**

Prior studies in the National Basketball Association (NBA) and Women's National Basketball Association (WNBA) reported worse player performance after Achilles tendon rupture (ATR).

**Purpose/Hypothesis:**

The purpose of this study was to compare time to return to play (RTP) and performance after ATR between NBA and WNBA athletes. It was hypothesized that there would be no relative difference between the NBA and WNBA players.

**Study Design:**

Cohort study; Level of evidence, 3.

**Methods:**

ATRs in the NBA between 1987 and 2017 and WNBA between 2006 and 2017 were identified through a rigorous online search of articles. Included athletes had no prior leg injuries and had played ≥3 seasons before and after ATR. Sex, age, position, body mass index, height, years of experience, time to RTP, and player efficiency rating (PER) were recorded. Relative performance was measured by matching injured athletes to uninjured controls in the same league in a 1:2 ratio. Relative differences were compared between leagues, with adjustment for baseline features. Multiple regression analysis was employed to identify variables correlating with RTP and PER.

**Results:**

Included were 102 professional basketball players, of whom 34 sustained ATR (21 male, 13 female). Sex/league correlated with differences in RTP (*P* < .001). There was a significant difference between the WNBA and NBA in PER when comparing 1 year pre- and 1 year postinjury (1.49 ± 0.25 vs 3.87 ± 0.43, respectively; mean ± SD *P* < .001). Compared with intraleague controls, the relative difference in PER postinjury was 0.81 ± 0.11 (WNBA) and 3.9 ± 0.89 (NBA) (*P* < .001). Multiple regression analysis indicated that when controlling for years of experience, player position, and age, NBA players took 126 days longer than WNBA players to RTP (*P* < .001) and NBA players had 9.96 times increased odds of taking >200 days to RTP compared with WNBA players (*P* = .006).

**Conclusion:**

Sex/league was a significant predictor of RTP after ATR. When compared with their respective controls, NBA players saw a greater decrease in postinjury performance than WNBA players. NBA players took longer to RTP than WNBA players. ATRs appear to more negatively affect NBA players than WNBA players.

Achilles tendon ruptures (ATRs) are most common in the sports setting, and they are more commonly seen in men than in women.^26,33^ Of all sports, ATR occurs most frequently in basketball.^4^ The mechanism of rupture involves a single heavy loading force on the ankle accompanied by sudden acceleration or deceleration.^2^ Professional basketball players experience this biomechanical loading several times per game, with ATR occurring most commonly from a noncontact mechanism during “take-off” from a stop with the foot dorsiflexed, knee in early flexion, and hip extended.^21^ At the professional level, ATR can be a career-altering injury, with a 70% to 90% successful return-to-play (RTP) rate and a reoperation rate of 3.3% among athletes.^28,30^ In the National Basketball Association (NBA), athletes who RTP after an ATR have decreased playtime and performance relative to the rest of the field in the season they return.^21^ Additionally, in the NBA, ATR occurred at a rate of nearly 1 per season between 1970 and 2018, with 36.8% of players either not achieving RTP or starting fewer than 10 games for the remainder of their career.^21^ Similarly, in the Women's NBA (WNBA), players who sustained an ATR were frequently able to RTP, but their career longevity was less than their peers.^3,31^

The literature comparing outcomes in professional male and female athletes after injury is sparse. The 2021 retrospective review by Glein et al^13^ of 73 professional and collegiate athletes who underwent primary hip arthroscopy between March 2009 and July 2018 found that female athletes achieved a minimal clinically important difference at higher rates than men on the Hip Outcome Score–Sport-Specific Subscale (*P* = .035) and the Nonarthritic Hip Score (*P* = .037). However, their study did not utilize player performance after surgery as an objective postoperative outcome, which is an important measure for these athletes.

The relative effects of ATR on NBA compared with WNBA players are not well characterized. The primary aim of this study was to identify differences in time to RTP and relative player efficiency ratings (PERs) between NBA and WNBA athletes who sustained ATRs. The secondary aim of this study was to analyze the relationship between demographic factors and recovery after ATR. We hypothesized there would be no difference between the NBA and WNBA athletes.

## Methods

We conducted a retrospective review of ATRs sustained by WNBA players between 2006 and 2017 and NBA players between 1987 and 2017. Players with ATRs were identified from news articles, injury reports, and official association websites (www.NBA.com and www.WNBA.com). Search terms included a combination of “NBA,”“WNBA,”“Achilles tendon rupture,” and “Achilles tendon injury.” All ATRs included were verified by ≥2 independent sources. Two team members (R.Y., A.L.) independently conducted the search protocol, blinded to each other's work; their findings were merged, and duplicates were eliminated, after which a third team member (R.J.W.) performed the search protocol again to confirm that all potential sources were covered. Players were excluded if they sustained the ATR before their professional career in the NBA or WNBA, had a history of major lower extremity injuries on either leg before the ATR, or did not play in the NBA or WNBA for 3 full seasons before and after injury.

**Figure 1.**
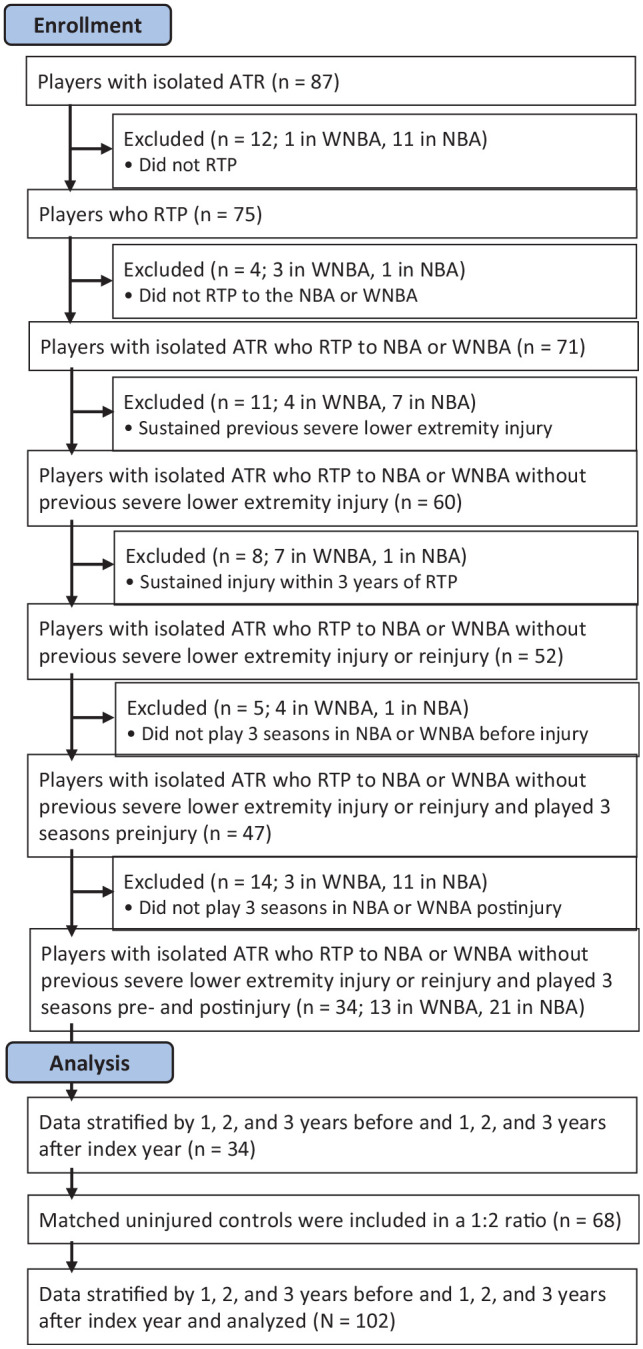
CONSORT (Consolidated Standards of Reporting Trials) diagram of players included in the study. ATR, Achilles tendon rupture; NBA, National Basketball Association; RTP, return to play; WNBA, Women's National Basketball Association.

Initially, 87 athletes were identified who had sustained an isolated ATR in the WNBA or NBA. Of these players, 54 did not meet the study inclusion criteria. Thus, 34 players who sustained an isolated ATR were ultimately included (13 WNBA, 21 NBA). Injured players were matched to uninjured control participants in a 1:2 ratio by league played, player position, body mass index (BMI), experience, age, and height. The season of the ATR was defined as the index year. Data were collected for each player for 3 years before the injury, labeled preindex (years –1, –2, and –3) and for 3 years after the injury, labeled postindex (years 1, 2, and 3). A graphical representation of this information can be found in Figure 1.

For all included participants, data on age, seasons played, height, weight, BMI, and position played were collected. RTP was determined using the first game in the NBA or WNBA played after ATR. Player performance data included seasons played, games played per season, minutes played per season, and PER. The PER is an overall rating of a player's per-minute productivity, calculated with the following formula: [*points*+*rebounds*+*assists*+*steals*+*blocks*+*turnovers*+*free throws made*+*field goals made – field goals attempted – free throws attempted*] ÷*games played*.

### Statistical Analysis

After data collection, the study sample was divided into 2 cohorts by sex (ie, league): those in the ATR and those in the control groups. All NBA athletes are male and all WNBA athletes are female; thus, the players included in the sex and league of the competition groups were identical. Microsoft Excel was used to sort and organize the data and perform preliminary analysis. The Statistical Package for the Social Sciences (SPSS; Version 28) suite (IBM) was utilized to analyze the data further.

Categorical results were reported as counts with column percentages. Continuous data were reported as means and standard deviations, with standard errors provided as appropriate. All data were first analyzed to ensure an apt statistical assessment was chosen. A comparison of normally distributed data was performed with independent- and paired-samples *t* tests. For nonnormally distributed data, the Wilcoxon rank-sum test was performed. Multiple linear and logistic regression models were analyzed to ensure all assumptions were met. Residuals were assessed for normal distribution, and no multicollinearity was observed. Furthermore, the data showed homoscedasticity. All variables in the multiple linear logistic regression model were first run separately to ensure no artifact *P* values were present and all effect sizes were reported with fidelity.

The change in PER (ΔPER) in each league was calculated by finding the difference between 1 year preindex and 1 year postindex (ΔPER_1y pre-post_). The same process was conducted to calculate the pooled difference in PER in each league for the 3 years preindex and 3 years postindex (ΔPER_3y pre-post_). The Student *t* test was then used to compare the ΔPER_1y pre-post_ of the WNBA ATR group and the NBA ATR group and the ΔPER_3y pre-post_ of the WNBA ATR group and the NBA ATR group.

Each ATR cohort underwent further analysis of preindex performance metrics compared with postindex and then postindex compared with controls using independent- and paired-samples *t* tests. Next, the data from both sex/leagues were pooled and analyzed based on sex as the primary independent variable. Multiple linear and logistic regression models were created to elucidate the connection between sex and RTP, while controlling for age, BMI, and player position. Last, a relationship between RTP and various other factors was further explored through the Pearson *r* and Cramér *V* coefficients.

Last, the G*Power statistics tool (Heinrich-Heine-Universität Düsseldorf) was used to perform power analysis for each group of injured players and their respective controls. It was also performed across male and female injured players. Confidence intervals were set at 95%, and *P* < .05 was considered statistically significant. Post hoc power analysis was executed with consideration of the Cohen *d* coefficient within independent- and dependent-samples *t* tests.

## Results

Of the 102 professional basketball players who comprised the patient pool for this study, 34 sustained an ATR (21 male [NBA players] and 13 female [WNBA players]), and 68 served as matched controls, which at a 1:2 ratio included 42 NBA players and 26 WNBA players. Thus, an overall total of 63 athletes who played in the NBA and 39 in the WNBA participated in this study. A total of 11 NBA players and 1 WNBA player did not achieve RTP; of these players, there were no significant differences in player position, years of experience, or age between leagues. The NBA group took significantly longer to RTP after ATR than the WNBA group (343 ± 118 vs 195 ± 81 days, respectively; *P* < .001) (Table 1).

**Table 1:** General Characteristics of All Athletes and Athletes With ATR by League^*a*^

Characteristic	All Athletes (N = 102)	Athletes With ATR (n = 34)		
		WNBA (n = 13)	NBA (n = 21)	*P*
Age, y	29 ± 4	31 ± 5	29 ± 4	.18
Height, cm	195 ± 14	183 ± 7	202 ± 10	**<.001**
Weight, kg	92 ± 17	77 ± 8	104 ± 18	**<.001**
BMI, kg/m^2^	24.1 ± 2.12	22.89 ± 1.59	25.46 ± 2.65	**.01**
Return to play, d	295 ± 127	195 ± 81	343 ± 118	**<.001**
Experience, y	8.05 ± 3.77	8.48 ± 3.95	7.81 ± 4.12	.65
Player position				.444
Center	27 (0.27)	5 (0.42)	3 (0.14)	
Forward	46 (0.47)	4 (0.33)	14 (0.67)	
Guard	26 (0.26)	3 (0.25)	4 (0.19)	
Reinjury	8 (0.23)	7 (0.53)	1 (0.05)	**<.001**

a Data are reported as mean ± SD or n (%). Boldface *P* values indicate statistically significant difference between groups as shown (*P* < .05). ATR, Achilles tendon rupture; BMI, body mass index; NBA, National Basketball Association; WNBA, Women's National Basketball Association.

There were no significant differences in demographic characteristics between the NBA ATR group and NBA controls. There was a significant difference in PER when comparing the NBA ATR group with their controls at 1 year postindex (ATR: 13.1 ± 3.4, control: 17 ± 4; *P* < .001) and 2 years postindex (ATR: 12.3 ± 4.3, control: 16.2 ± 3.8; *P* < .001) (Table 2). However, there was no significant difference in PER between the NBA ATR group and NBA controls 3 years postindex. No significant difference in demographic characteristics was identified when comparing the WNBA ATR group with WNBA controls. Additionally, no significant difference in PER was identified in the 3 years before injury when comparing injured WNBA players with their uninjured controls and when comparing injured NBA players with their uninjured controls. In the WNBA ATR group, there was no significant difference in PER when returning from injury compared with WNBA controls (Table 2). When comparing the difference in control PER and ATR PER (ΔPER_control–ATR_) by sex, the male (NBA) players showed a markedly larger drop in performance both in the year immediately after (3.9 ± 0.89 vs 0.81 ± 0.11, respectively; *P* < .001) and in the 3 years of pooled data (3.27 ± 0.56 vs 0.17 ± 0.08, respectively; *P* < .001) (Table 2). However, the female (WNBA) players had a markedly higher proportion of reinjury within the 3 postindex years.

**Table 2:** General Characteristics and Performance Metrics by Control and ATR Group Within Each League^*a*^

Characteristic	WNBA			NBA		
	Control	ATR	*P*	Control	ATR	*P*
Return to play, d	—	195 ± 81	—	—	343 ± 118	—
Experience, y	8.11 ± 3.64	8.48 ± 3.95	.78	8.01 ± 3.75	7.81 ± 4.12	.84
Age, y	28 ± 3	31 ± 5	.06	28 ± 3	29 ± 4	.58
Height, cm	182 ± 8	183 ± 7	.59	202 ± 11	202 ± 10	.91
Weight, kg	75 ± 8	77 ± 8	.53	100 ± 12	104 ± 18	.31
BMI, kg/m^2^	22.8 ± 1.76	22.89 ± 1.59	.88	24.52 ± 1.52	25.46 ± 2.65	.08
Career, y	11.73 ± 3.42	11.35 ± 4.12	.779	14.36 ± 2.79	12.50 ± 3.98	**.039**
PER preindex						
3 y	14.6 ± 4.1	16.7 ± 4.9	.2	16.9 ± 4.9	16.3 ± 4.1	.69
2 y	14.6 ± 3.6	15.1 ± 5.1	.75	17.4 ± 3.9	16.6 ± 5.5	.5
1 y	14.91 ± 4.45	15.38 ± 5.92	.79	17.29 ± 4.08	16.97 ± 3.84	.38
PER postindex						
1 y	14.7 ± 3.7	13.89 ± 4.9	.11	17 ± 4	13.1 ± 3.4	**<.001**
2 y	12.8 ± 5.1	13.5 ± 4.9	.66	16.2 ± 3.8	12.3 ± 4.3	**<.001**
3 y	13.6 ± 6.1	13.2 ± 7.1	.84	15.5 ± 4.7	13.5 ± 2.7	.12
ΔPER_control–ATR_						
1 y	WNBA: 0.81 ± 0.11, NBA: 3.9 ± 0.89 (***P* < .001**)					
Pooled 3 y	WNBA: 0.17 ± 0.08, NBA: 3.27 ± 0.56 (***P* < .001**)					

a Data are reported as mean ± SD. Dashes indicate areas not applicable. Boldface *P* values indicate statistically significant difference between groups as shown (*P* < .05). ATR, Achilles tendon rupture; BMI, body mass index; NBA, National Basketball Association; PER, player efficiency rating; WNBA, Women's National Basketball Association.

**Figure 2.**
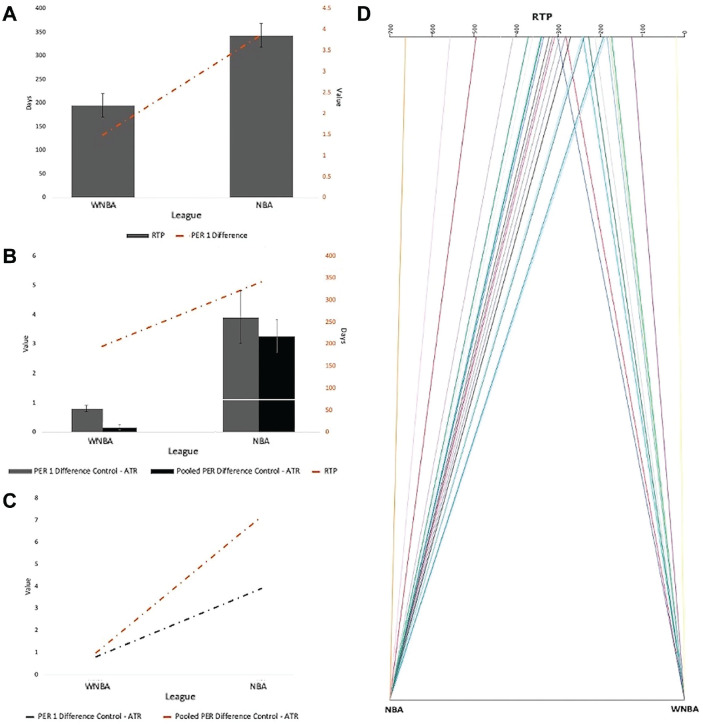
Comparison of players by league. Error bars in (A) and (B) indicate SE. (A) Dual plot of RTP and ΔPER_1y pre-post_. Injured NBA players had a longer RTP duration than their WNBA counterparts (343 days [SE, 25.7 days] vs 195 days [SE, 25.7 days]) and a higher ΔPER_1y pre-post_ (3.87 [SE, 0.43] vs 1.49 [SE, 0.25]), *P* < .001 for both. (B) Triple plot of RTP, ΔPER_1y control-ATR_, and ΔPER_3y control-ATR_. NBA players had a longer RTP duration, higher ΔPER_1y control-ATR_ (3.9 [SE, 0.89] vs 0.81 [SE, 0.11]), and higherΔPER_3y control-ATR_ (3.27 [SE, 0.56] vs 0.17 [SE, 0.08]), *P* < .001 for all. (C) Dual line plot of ΔPER_1y control-ATR_ and ΔPER_3y control-ATR_. NBA players had a higher ΔPER_1y control-ATR_ (3.9 [SE, 0.89] vs0.81 [SE, 0.11]) and higher ΔPER_3y control-ATR_ (3.27 [SE, 0.56] vs 0.17 [SE, 0.08]), *P* < .001 for both. (D) Parallel flow plot of RTP with respect to league. NBA players had a significantly longer mean RTP duration than their WNBA counterparts (343 [SE, 118] vs 195 [SE, 81]), *P* < .001. ATR, Achilles tendon rupture; NBA, National Basketball Association; PER, player efficiency rating; Pooled PER, PER_3y;_ pre-post, pre– or post–index year; RTP, return to play; WNBA, Women's National Basketball Association.

Within the NBA ATR group, there was a significant decrease in PER when comparing the 1 year preindex (16.97 ± 3.84) with the 1 year postindex (13.10 ± 3.40) (*P* < .001). Additionally, there was a significant decrease when comparing pooled PER for 3 years preindex (16.59 ± 4.37) with pooled PER for 3 years postindex (13.77 ± 2.88) (*P* < .001) within the NBA ATR group (Table 3). Within the WNBA ATR group, no significant change in PER was identified when comparing pre- and postindex performance (Table 3). A significant difference in the ΔPER_1y pre-post_ was found between the WNBA ATR (1.49 ± 0.25) and NBA ATR (3.87 ± 0.43) groups (*P* < .001). However, no significant difference was identified when comparing the ΔPER_3y pre-post_ in the WNBA ATR group and NBA ATR group (Table 3 and Figure 2).

**Table 3:** Within-Group Comparisons of Pre- Versus Postindex Year by League^*a*^

PER	WNBA		NBA	
	Value	*P*	Value	*P*
1 y before and after index year				
Pre	15.38 ± 4.99	.05	16.97 ± 4.00	**<.001**
Post	13.89 ± 4.22		13.1 ± 4.23	
Δ	1.49 ± 0.25		3.87 ± 0.43	**<.001**
3-y pooled data before and after index year				
Pre	15.96 ± 4.92	.05	16.59 ± 4.37	**<.001**
Post	13.69 ± 5.33		13.77 ± 2.88	
Δ	2.27 ± 0.83		2.82 ± 0.42	.053

a Data are reported as mean ± SD. Boldface *P* values indicate statistically significant difference between preindex (Pre) and postindex (Post) (*P* < .05). NBA, National Basketball Association; PER, player efficiency rating; WNBA, Women's National Basketball Association.

**Figure 3.**
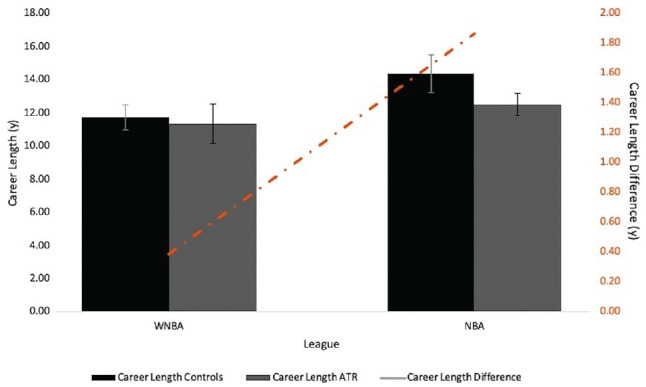
Triple plot of career length in controls and injured athletes (Achilles tendon rupture [ATR]) within each league. The line represents the mean career length difference between the controls and ATR within each league. Injured WNBA players did not have a significant difference in their mean career length when compared with their matched controls, 11.35 years (SE, 0.74 years) vs 11.73 years (SE, 1.20 years), respectively (*P* = .779). NBA players had a significant difference in their mean career length when compared with their matched controls, 12.50 years (SE, 1.14 years) vs 14.36 years (SE, 0.65 years), respectively (*P* = .039) . NBA, National Basketball Association; WNBA, Women's National Basketball Association.

NBA/WNBA league participation, interchangeably defined as athlete sex, was the only study variable found to correlate significantly with RTP (*V* = 0.65; *P* < .001) (Table 4). BMI, height, weight, age, years of experience, and player position were not significant predictors of RTP within either the WNBA ATR group or the NBA ATR group, nor were they predictive of RTP in the combined sample. Players in the WNBA ATR group had a career length equal to that of their controls, but there was a significant decrease in the mean career length of players in the NBA ATR group (12.50 years; SE, 1.14 years) compared with NBA controls (14.36 years; SE, 0.65) (*P* = .039), as can be seen in Figure 3.

**Table 4:** Correlation of Characteristics With RTP^*a*^

Characteristic	Cramér *V*	*P*
League	0.65	**<.001**
Experience	−0.16	.38
Age	−0.31	.09
BMI	0.21	.26
Height	0.27	.14
Weight	0.28	.13
Player position	0.03	.88

a Boldface *P* value indicates statistical significance (*P* < .05). BMI, body mass index; RTP, return to play.

Multiple linear regression found that when controlling for years of experience, player position, and age, the NBA ATR group took 126.24 days longer to RTP than the WNBA ATR group (*P* < .001). Multiple logistic regression found that the NBA ATR group had 9.96 times increased odds of taking >200 days to RTP than the WNBA ATR group when controlling for years of experience, player position, and age (*P* = .006).

## Discussion

We had hypothesized there would be no sex-based differences between NBA and WNBA athletes with regard to RTP and PER after ATR. However, we found that (1) NBA players took almost twice as long as WNBA players to RTP; (2) upon RTP, NBA players saw a greater decrease than WNBA players in personal postinjury performance, as well as relative performance compared with controls; and (3) when controlling for age and years of experience, the sex of the athlete (and hence league of competition) significantly predicted time to RTP.

In professional athletes, ATR is treated surgically. Postoperatively, the American Orthopaedic Society for Sports Medicine (AOSSM) recommends a 6- to 8-week period of tendon protection with a cast or brace, followed by a rehabilitation program to regain function for 6 months, after which patients can return to athletic activities, assuming appropriate rehabilitation progressions.^16^ Our study found that time to RTP was 195 days in the WNBA versus 343 days in the NBA. While WNBA players followed the AOSSM protocol timeline closely, NBA players took almost twice as long. We suspect that physiologic differences alone do not explain these differences. Some literature suggests that men might be expected to recover faster than women. Women have a lower rate of new connective tissue synthesis and have lower rates of acute collagen synthesis after exercise than men.^24^ Furthermore, female patients have shown a greater relative deficit in heel-rise height when compared with male patients after ATR. Therefore, other factors must account for the prolonged RTP in men compared with women.

Determining underlying reasons for differences in NBA versus WNBA athletes’ RTP after ATR goes beyond the scope of the present study. The differences we found were unexpected. There may be factors other than the sex of the athletes competing in professional basketball that account for the observed differences between the leagues. One potential factor may be financial; the mean salary of players in the WNBA in the 2017-2018 season was $75,000, whereas the mean salary of players in the NBA was $6,100,000.^1,23^ NBA players may thus be incentivized to be more cautious about RTP as they can face far more substantial financial losses if they become reinjured. It is also possible that the relatively greater monetary resources of the NBA factor into how teams and athletes approach rehabilitation with regard to training room, physical therapy, and RTP progressions, as the NBA is a wealthier league.^1^ In both leagues, injured players are paid through their injuries as defined by their contract, meaning that WNBA players do not face financial pressures to RTP more quickly for fear of losing salary.^23^ Additionally, play in the NBA is more centered around explosive individual play than in the WNBA, which heavily emphasizes the technical and team aspects of basketball.^6^ This increased need for individual athleticism in the NBA could contribute to the longer time to RTP. Furthermore, the WNBA has substantially fewer players than the NBA; having fewer substitute or backup players available may create pressure for faster RTP. Future research may help identify factors that underpin the observed differences in NBA versus WNBA RTP after ATR.

The frequency of ATRs in our study also differed significantly by league. We found that WNBA players sustained ATR at a rate of 1.08 per season, while NBA players sustained ATR at a rate of 1.00 per season (*P* < .05). This is consistent with the findings from the systematic review of Lian et al^22^ in 2022, which found a mean 1.1 ATRs per season in the WNBA between 2000 and 2019 and 1.0 per season in the NBA. In addition to physiology, there are differences in male and female biomechanics. During jumping, there are various ankle landing angles that may overload or compromise the Achilles tendon. Karabeg et al^18^ created a mathematical model of landing angles for male athletes and found a critical point of approximately 36° that generated force transfers of tendon overload; additionally, they discussed the use of footwear in mitigating landing angles after jump shots. Although a similar female study does not exist, there are known differences in the musculoskeletal architecture and biomechanics, and the role of footwear remains unclear. Firminger et al^12^ created musculoskeletal models from 30 male basketball players wearing 3 different shoes that varied in stiffness and found significant differences in Achilles tendon strains and changes in ground-reaction forces with the least stiff shoe reducing the strain on the Achilles tendon without affecting performance. Data on the type of footwear female professional athletes use are lacking. It is possible that male-centric design principles are incorporated into commercially available basketball shoes for women and that these designs are suboptimal for female Achilles tendon biomechanics.

The impact of ATR on player retirement was not assessed by our study. However, this has been assessed in previous studies and warrants mention for a better understanding of economic forces that may influence RTP. Alegre et al^4^ found that after sustaining ATR, 36.8% of NBA players had to retire or played in <10 games for the rest of their professional career. Trofa et al^32^ found that ATR prevented RTP of 30.6% of professional athletes when aggregating across the NBA, National Football League, National Hockey League, and Major League Baseball. Amin et al^5^ found that 39% (7 out of 18) of players sustaining ATR in the NBA between 1988 and 2011 did not RTP. In the WNBA, Hodgens et al^14^ found in their sample of 17 players that sustained ATR between 2000 and 2019, 23.5% of players did not RTP, and an additional 41.2% of players did not remain in the league after 1 season. Tramer et al^31^ found in their sample of 12 WNBA players between 1997 and 2019, 16.7% (2 players) of players did not RTP. In a retrospective review of 12 WNBA players between 2006 and 2018, Federico et al^11^ found that 42% (5 players) of players did not RTP. There are large discrepancies in what the rate of RTP after ATR is, especially in the WNBA. A comprehensive database of professional basketball players who sustain ATR could potentially more accurately assess the rate of RTP.

Although basketball is a contact sport, video analysis of 12 ATRs in the NBA revealed an absence of contact during injury.^21^ It follows that the forces required to rupture the Achilles tendon are the product of solely the patient's generated forces and ground landing forces. Therefore, the relationship between tendon integrity, self-generated forces, and landing angles is an important factor in ATR. Achilles tendons are anatomically thinner in female athletes. A review found that, by adolescence, male athletes had developed thicker Achilles tendons than female athletes.^7,17^ Self-generated forces in women are less than those of men secondary to decreased muscle mass; however, flexibility is reduced in male athletes, which affects the athlete's ability to make microadjustments before landing and thus to potentially avoid injury.^34^

Although this is the first study to compare sex-based differences in time to RTP in professional athletes after ATR, sex-based differences in recovery after ATR has been previously studied. Silbernagel et al^27^ conducted a retrospective review of 182 patients sustaining ATR (152 male, 30 female), between the ages of 20 and 70 years, and found that female patients had a greater deficit in heel-rise height after recovery from ATR than their male counterparts even when controlling for surgical versus nonsurgical treatment. However, their study considered the general population and included patients that were treated nonsurgically. Professional athletes tend to be treated surgically after ATR and are generally younger than the patients included in their review. Our study is unique in that it pertains to performance in professional athletes, where performance after an ATR is critical because it is foundational to their livelihood. In addition, several studies have examined sex-based differences in outcome scores and postoperative pain levels as well as rates of injury. In a cohort study, Daniels et al^8^ found that while women had higher pain scores in the first 3 months after arthroscopic rotator cuff repair, there were no significant differences at 1-year follow-up. Furthermore, in elective total hip arthroplasty, female patients were more likely to report severe pain and worse outcomes.^15,25^ These findings are contrary to those of the female athletic population, where Glein et al^13^ demonstrated that female athletes undergoing hip arthroscopic surgery have a greater improvement in outcome scores and pain control. Karlson et al^19^ explained that differences may be attributed to preferences and readiness to perform elective procedures of the patients. Moreover, in recovery after sports-related concussions, Stone et al,^29^ in a retrospective review of 579 athletes (365 male, 214 female) in middle school, high school, and college, found that female athletes took significantly longer to start an RTP progression after a sports-related concussion than age-matched male athletes (*P* = .002). Additionally, Davis-Hayes et al^9^ conducted a 15-year retrospective cohort study of 1200 varsity collegiate athletes (822 male, 378 female) and found that female athletes were more likely to sustain a sports-related concussion (*P* = .01). However, that study did not find a difference in time to RTP across sex when controlling for the presence of postconcussion symptoms.^9^

We found that there was a markedly high rate of reinjury within the WNBA cohort. One potential reason for this is that female professional basketball players in the WNBA have a significantly higher rate of lower extremity injuries per 1000 athlete-exposures, according to a retrospective injury comparison study between female and male athletes (14.6 vs 11.6) (*P* < .05).^10^ Though we did not perform an analysis on primary injury rates, our markedly higher reinjury rates within the WNBA cohort are consistent with the higher rates of injury in the female leagues reported by Dietch et al.^10^ Moreover, the increased reinjury rate aligns with findings from Knobloch et al,^20^ demonstrating diminished effectiveness of eccentric training for alleviating symptoms of midportion Achilles tendinopathy in female patients compared with male patients. Investigating differences between male and female athletes after injuries could further inform sex-specific treatment approaches, enhancing recovery outcomes. Additionally, it is crucial to consider potential social and economic pressures influencing the decision to RTP.

### Limitations

This study has limitations. The retrospective nature of this study predisposes to bias in data retrieval and interpretation. Additionally, the overall incidence of ATR is low. Professional athletes have access to extensive rehabilitation and expert medical staff, as well as time to focus on rehabilitation after injury or surgery, which limits the generalizability of these results to less elite athletes and the general population. Another potential limitation of our study is the difference in the time period utilized for the NBA and WNBA players. The WNBA was established in 1996, while the NBA started in 1946. NBA players were included in 1987, as online reports of these players’ injuries could be found beginning that year. Records of injuries in the WNBA before 2006 were not available. This study would be improved upon by having a total database of injuries for the entire existence of both leagues. The low incidence of ATR means that data collection must go back in time to obtain statistically meaningful patient numbers. While the concept of open surgical repair today remains similar to past decades, rehabilitation modalities and techniques have evolved, and it is likely that a number of other variables such as augmentation with biologics, attention to athlete nutrition, and other factors that might affect outcome and RTP are not captured in our analysis. Furthermore, up to 30% of professional athletes sustaining ATR do not RTP, and to allow for performance comparisons, we did not include players in our study that did not RTP. However, we excluded far more NBA than WBNA athletes for that reason, so we believe this was not a major factor in our analysis.

It is also worth noting that as public news sources were the primary reporting channels for these injuries, it was often not feasible to obtain detailed information on each player's specific operative or nonoperative management. Consequently, variations in factors such as surgical method, surgery timing, and rehabilitation concerns—such as cast duration at different plantarflexion angles—among other factors could exist between the leagues. The players were also identified primarily using publicly available news articles, predisposing to inaccurate injury identification. However, confirming injuries using 2 independent news sources and utilizing player statistics aided in confirming those players who were truly injured.

## Conclusion

Player sex/league was a significant predictor of an athlete's RTP after ATR. NBA players took longer to RTP than WNBA players. When compared with their respective controls, NBA players saw a greater decrease in postinjury performance than WNBA players. ATRs appear to more significantly negatively affect NBA players than WNBA players.
